# Identifying the main barriers for participation in a population-based colorectal cancer screening programme in East Azerbaijan, Iran

**DOI:** 10.3332/ecancer.2022.1354

**Published:** 2022-02-10

**Authors:** Roya Dolatkhah, Mohammad Hossein Somi, Saeed Dastgiri, Mohammad Asghari Jafarabadi, Bita Sepehri, Masoud Shirmohammadi, Marzieh Nezamdoust, Hossein Mashhadi Abdolahi, Faris Farassati

**Affiliations:** 1 Hematology and Oncology Research Center, Tabriz University of Medical Sciences, Tabriz, Iran, PO Box: 5166614731; 2 Liver and Gastrointestinal Diseases Research Center, Tabriz University of Medical Sciences, Tabriz, Iran, PO Box: 5166614756; 3 Tabriz Health Services Management Research Center, Tabriz University of Medical Sciences, Tabriz, Iran, PO Box: 5166615739; 4 Department of Statistics and Epidemiology, School of Medicine, Zanjan University of Medical Sciences, Zanjan, Iran and, Center for the Development of Interdisciplinary Research in Islamic Sciences and Health Sciences, Tabriz University of Medical Sciences, Tabriz, Iran, PO Box: 5463789200; 5 Liver and Gastrointestinal Diseases Research Center, Tabriz University of Medical Sciences, Tabriz, Iran, PO Box: 5166614756; 6 Tabriz Health Services Management Research Center, Tabriz University of Medical Sciences, Tabriz, Iran, PO Box: 5165990001; 7 Midwest Biomedical Research Foundation, Kansas City, MO 64128, USA; a https://orcid.org/0000-0002-6897-7120; b https://orcid.org/0000-0002-6611-9958; c https://orcid.org/0000-0002-8129-9125

**Keywords:** screening, barrier, colorectal cancer

## Abstract

**Background:**

Colorectal cancer (CRC) is the third most common cancer and the second leading cause of death worldwide. However, CRC is considered as one of the most preventable cancers by which the mortality rates reduce about 60% through implementing the screening programmes. The present study aimed to evaluate the main barriers of CRC screening in a defined population.

**Method:**

Healthy individuals from all regions of the state were invited to participate in different healthcare centres. They were assessed by a provided online risk assessment tool, which was completed for all recruited subjects, and has been developed to assess the CRC risk based on personal and family history of adenoma, CRC, and other high-risk diseases. Research team staff assessed all individuals by this tool and then eligible people according to their lifetime risk of CRC were included in the study. There was not any age restriction in this study. Colonoscopy and three stool-based tests including faecal occult blood test, faecal immunochemical test and stool DNA tests were performed.

**Results:**

Overall, 725 cases including 425 (58.6%) males and 300 (41.4%) females participated in the study. Lack of knowledge and attitude about screening programmes was the most common barrier, especially among women (68% for women versus 58% for men) and those from rural areas (88% in rural versus 55% in urban areas). Fear of colonoscopy and procedure complications and pain (48%), discomfort and anxiety from inserting a tube into the bowel (65% among females versus 43% among males) were reported commonly. Embarrassment and dignity were other complaints, especially in women (62% in females versus 35% in males).

**Conclusion:**

Increasing knowledge and attitude about the aims and benefits of screening programmes, acceptable and convenient communication of health systems with the general population are considered to be the key elements in the success and implementation of any screening programme.

## Introduction

Colorectal cancer (CRC) was the third most common cancer and the second leading cause of death from cancer during 2020 in the world [1]. In addition, CRC ranked the third most common cancer in Iran, but second in East Azerbaijan, with an increasing trend of annual percentage changes, 7.6% per year during the last 12 years [2–4]. CRC is considered to be one of the most preventable cancers and population-based screening programs can reduce mortality by up to 60% [5–7]. Some evidence confirmed a decrease in the burden of disease in terms of incidence and mortality by implementing screening and early detection programmes [8, 9]. Despite the recommendations and guidelines provided by organisations and committees for screening and early detection of CRC, mass screening has not yet taken place at the population level in many countries [8]. Even in the developed countries like the United States, only about 61% of age-eligible people were currently screened, and there are still many barriers to the comprehensive implementation of CRC screening and people are much less likely to be screened than expected [8–11]. There are limited organised screening programmes in Iran by focusing on risk assessment and risk reduction guidelines. Some evidence and studies were reported from local and regional CRC screenings, and some limited studies have focused on the barriers of CRC screening in Iran [12–16].

Barriers to CRC screening can be considered in two main categories:

(1) Personal and/or procedural related factors or the so-called Patient Related Factors: They are related to a lack of public awareness about the goals and benefits of CRC screening programme. Fear and discomfort related to colonoscopy, pain during the procedure, cancer, bowel preparation difficulties, complications ranked as the most important and common causes reported in a large number of studies [11, 17–19].

(2) Practical and/or health system problems or the so-called System Related Factors: The most important problems of this category are the lack of awareness of primary healthcare (PHC) workers and physicians, misinformation about colonoscopy and its possible harms, crowded public and referral centres for screening and cost and length of colonoscopy. Lack of availability of screening tests in all treatment centres and scheduling appointment are regarded as other problems [18, 19].

However, regarding CRC screening, there is no definitive recommendation for choosing the golden standard technique and the best screening method for the average risk population. In addition, there is still no consensus between communities and organisations [20, 21]. According to the latest National Comprehensive Cancer Network (NCCN) guideline, colonoscopy is now recommended as the first method for average risk people, every 10 years with the highest sensitivity (95%) and specificity (90%) for CRC detection. Other alternatives are stool-based tests including high sensitivity guaiac-based test (faecal occult blood test (FOBT)), immunochemical based test (faecal immunochemical test (FIT)), stool DNA test, flexible sigmoidoscopy and computerized tomographic (CT) colonography [22].

The results of the present study are in line with those in the previous study regarding the development and validation of CRC risk assessment tool by using translated and validated NCCN guideline [23], in order to introduce and implement a CRC screening programme in East Azerbaijan province. In this programme, colonoscopy and three stool-based tests including FOBT, FIT and stool DNA test were performed for all eligible people. However, due to many problems and barriers during the study, mainly in the case of colonoscopy for eligible candidates, this section of the study was separately discussed. In other words, to the present study aimed to assess and report the main barriers of colonoscopy in implementing the population-based CRC screening in East Azerbaijan.

## Material and methods

### Research design

The present study aimed to establish the CRC screening programme in East Azerbaijan Province during 2016–2019. This study was designed in two phases. First, the screening study was done as a pilot phase to assess the research feasibility in the context of one of the most important and successful cohort programmes in the country, Azar Cohort. This cohort study was established in East Azerbaijan and covered 15,000 of 35–70 years old population of Shabestar city and its counties. The reason for choosing this community was the good performance and follow-up by the well-trained staff about the programme and good cooperation of the registered population in this area. The estimated sample size was 500 people during the first phase of the study (2016).

In the second phase, we cooperated and contacted healthcare centres, Educational and Treatment Centres, Clinics and charities in the whole province by official letters, advertisements and telephone calls in order to invite all eligible people from the whole province. In addition, a team of well-trained and interested research staff and medical students encouraged eligible individuals to participate in the screening programme. They followed the individuals through telephone calls and social media and introduced the objectives of the study to encourage people to participate, as well as giving complete information about the outcomes and benefits of the screening programmes. The estimated sample size was 500 people during the second phase of study (2017–2019).

### Study subjects

East Azerbaijan is a province located in Northwest of Iran, which has the sixth largest population and the most Azeri ethnic population in Iran. In addition, it covers an area of 45,620 km^2^ and has a total population of 3,911,278 according to the last national census in Iran. Like other provinces, East Azerbaijan and Tabriz University of Medical Sciences benefit from a well-established PHC network. The network is well-organised and is credited with the improvements in health outcomes observed since the 1980s.

### Sample size determination

The total sample size for the study has been estimated at 1,000, with an estimated prevalence rate of 5%, sensitivity of 80%, specificity of 80%, precision of 10% and with 95% confidence interval, using Stata MP 14.2 (StataCorp LP, College Station, TX 77845, USA) software.

### Intervention

Colonoscopy and three stool-based tests including FOBT, FIT and stool DNA test were performed for all eligible participants from the two phases. They were followed by online Risk Assessment Tool and filled questionnaire, or called them for their mentioned problems and barriers. Colonoscopy was performed in four referral hospitals and by ten expert gastroenterologists. Stool-based tests were performed in Hematology Lab and Immunology Research Center of Tabriz University of Medical Sciences.

The research team provided some facilities for the participants. The facilities included scheduling colonoscopy appointment from four referral endoscopy centres as the preference (gender, time, place), providing drugs for bowel preparation, their re-contacting the day before the colonoscopy and reminding the colonoscopy time, and tracking the use of laxative drugs, transferring to the colonoscopy centre, tracking the colonoscopy results and following the pathological results if the person had any biopsies of suspected lesions during the procedure.

### Measurements

All volunteers were evaluated through an online risk assessment tool. This tool was the translated and validated NCCN guideline which included a questionnaire with simple and easy-to-use questions about main risk factors of CRC [23]. Further, it was a user-friendly online system and risk assessment application (www.riskassessment.ir). This online tool provides a life time risk of CRC, and then gives recommendations for starting the best screening modalities for volunteers. A box was provided where participants could add their comments and suggestions, ask their questions and problems as a barrier for CRC screening. In fact, individuals reported their fears and personal barriers as open-ended discussions and received the necessary guidance. There was not any age restriction in this study, research team staffs assessed all individuals and then eligible people were included in the study.

All the data regarding patients’ barriers and complaints were collected from all eligible participants, even from those who refused to participate. These data were obtained online and by phone, as well as through personal follow-up and compliance. The questionnaire was designed and implemented according to researchers’ aims and scopes as a self-administered, anonymous and easy-to-answer tool, and using references. The response options designed as Likert scales and each variable scored from the best to worse status. They were asked to respond to a 5-point scale (strongly agree, almost agree, neutral, almost disagree, strongly disagree). Then, the scores were converted to 0–100 scales. The cut-points were poor for 1–5, moderate for 6–10, good for 11–15 and perfect for >15 scores.

In addition, an open-ended question was provided to allow the patients to describe any barrier they deemed important in Persian language. Their wording was later examined to develop the comprehensive CRC screening barriers.

### Laboratory analysis

Stool-based tests were performed in Hematology and Oncology and Immunology Research Centers of Tabriz University of Medical Sciences, including traditional guaiac-based FOBT (gFOBT), high-sensitivity guaiac-based FOBT (gFOBT Hb), FIT and multitarget stool DNA (Mt-sDNA) panel test.

The Mt-sDNA test consisted of molecular assays for mutant KRAS and BRAF, aberrantly methylated Bone Morphogenetic Protein 3 gene (BMP3) and N-Myc Downstream-Regulated Gene 4 (NDRG4) promoter regions, including aberrant methylation in the promoter regions of the ‘NDRG4’ gene and ‘BMP3’ gene and β-actin (a reference gene for human DNA quantity).

### Statistical analysis

Further, information during the colonoscopy was collected and recorded in a formal database. All print reports of the colonoscopy screening and/or pathology reports were sent to the research team. A comprehensive review and checking of the data were performed by a research team. All of the data were entered in an Excel file and rechecked with principal investigator (RD) again. Descriptive analysis was performed using Stata MP 14.2 (StataCorp LP, College Station, TX 77845, USA).

### Ethical clearance

This study was reviewed and approved by the ethics committee of the Tabriz University of Medical Sciences (ID: IR.TBZMED.REC.1395.635). Ethical consents were obtained from all the participants and all information and results were confidential.

## Results

**Figure 1.**
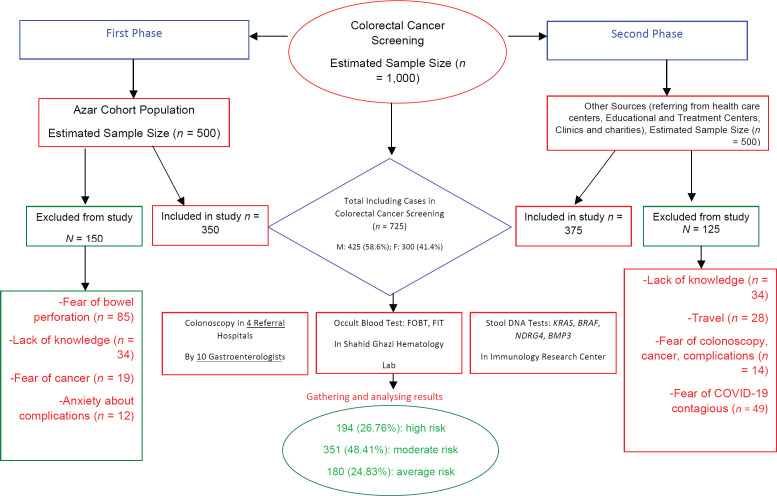
Population based CRC screening in East Azerbaijan flow-chart.

After facing with different barriers and problems, 150 cases were excluded from the study in the first phase and 350 were included. In the second phase, 375 cases were included and 125 people were removed due to some main barriers (Figure 1). Finally, 725 cases including 425 (58.6%) males and 300 (41.4%) females participated in this study and underwent CRC screening by colonoscopy and stool-based tests. Further, 442 (61%) were ≥50 years old. About 60% of 437 participants (*n* = 437) were related to rural areas and 40% (*n* = 288) were from urban areas. Nearly 52% of the participants (*n* = 374, 51.6%) had high education (Undergraduate, University); 215 (29.6%) had moderate education (high school) and 136 (18.8%) had low education (primary school or illiterate) (Table 1).

Overall, 194 (26.8%) participants were recognised as high risk people, 351 (48.4%) in moderate risk and 180 (24.8%) in average risk group. The most common barriers and complaints among 725 participants (Table 2) and 275 non-participants (Figure 1) are presented in two categories.

### Patient-related factors

Among non-participants, the most obvious problem was related to personal factors, while we failed to overcome and manage them. Figure 1 displays these problems in details. Among 150 cases who were dropped out of the study in the first phase, fear of bowel perforation (*n* = 85), lack of knowledge (*n* = 34), fear of cancer (*n* = 19) and anxiety about complications (*n* = 12) were the most important barriers leading to the withdrawal of volunteers. Among 125 non-participants in the second phase, the most reported barriers were lack of knowledge (*n* = 34), travel (*n* = 28), fear of colonoscopy, cancer and complications (*n* = 14) and fear of exposure to Coronavirus Disease 2019 (COVID-19) (*n* = 49).

Among all 725 participants, barriers and problems were collected and reported as well, however we overwhelmed them. The most common factor was related to lack of knowledge about screening programmes (68% in women versus 58% in men). Most of the people did not have any information about the significance and advantages of screening (62% in women versus 48% in men). Many people believed that screening is not required because of having no symptom, which was most obvious in males than females (67% in men versus 54% in women). In general, participants had little information about the preventability and treatability of CRC, which was more prevalent in women (54% in women versus 35% in men).

Another problem was related to the fear of colonoscopy and its complications such as bowel perforation, pain and bleeding during procedure and further hospitalisation (48% in female versus 35% in male). Further, anxiety about going to health centres (24% in men versus 37% in women) and fear and discomfort from inserting a tube into the bowel were common (65% in women versus 43% in men). Furthermore, embarrassment and dignity were considered as the most common issue in the cases (62% in females versus 35% in males). Regarding the differences between males and females, women mostly preferred to have their colonoscopy by a female doctor, even wait for scheduling for several weeks. However, this issue was more pronounced in the region as the community was Muslim with special religious beliefs.

Additionally, the problem related to taking laxative, bowel preparation medications and liquid diet for 3 days was mentioned as other barriers, which was reported differently in men and women. Men failed to follow the 3-day diet and laxative intake (36%), mainly because of disturbing their working hours. Women were more upset by the problem of pretest fasting and sedation during colonoscopy (28%).

Rural residences had less information about the need for CRC screening than urban residents (12% for rural versus 45% for urban). However, urban residents, especially highly educated people had more information about how to perform colonoscopy, pain and possible complications. Thus, they were more concerned and anxious than rural people about the procedure (15% for rural versus 54% for urban).

About 45% of the volunteers were concerned about fear of cancer, risk of any pre-malignant lesions, polyps and any positive findings during the colonoscopy. Lack of knowledge about prevention aims of screening programmes and decreasing CRC incidence and mortality by removing precancerous lesions and adenomas were considered as the common barriers and worries.

### Factors related to practical and health system facilitators

Most participants complained that no health centre or doctor was previously advised to have a screening and colonoscopy, and were completely unaware of the importance and outcomes of CRC screening.

A time-consuming and lengthy procedure was considered as another major barrier for people, which were most highlighted among men (48%). Regarding women, they were more anxious and worried about annoying any accompanying person (husband or family members) because they had to take sedative medications during colonoscopy, or take care of them after procedure (23%). In some men, scheduling and even referring to treatment centres were regarded as the main in some cases. However, they believed that colonoscopy is both faster and better (26% in male and 35% in female) when it is performed in a private clinic. On the other hand, some people believed that colonoscopy can be performed by residents or fellows at public educational and treatment centres, but they will definitely have a doctor who is sub-specialised in colonoscopy when they go to private centres. Thus, they preferred going to a private centre. The complication and adverse effect of colonoscopy were considered as another main barriers. In this regard, we faced with just one complication as bowel perforation in the first phase of the study, which led to complete cessation, and we missed 150 cases in this phase.

Lack of knowledge about the objectives and benefits of colonoscopy screening in staff and health-workers among endoscopy centres, and providing misinformation about colonoscopy and its possible harms increased the fear and anxiety of participants in some cases leading to their withdrawal (15% in men versus 22% in women). Travelling from hometown to colonoscopy centres which can add extra cost and time is regarded as another common problem among the participants. This problem was more difficult in women than men (47% versus 23%). Finally, it was reported that 24% of the people were unable to pay the travel costs.

## Discussion

The cornerstone of success and implementation of any cancer screening programme is increasing the public awareness about the benefits, aims and outcomes of screening. Thus, it is necessary to reduce the burden of disease and mortality by providing sufficient evidence by PHC workers and physicians to general population. However, facilitating the performing of screening programmes and providing the best, simplest and cost-effective modality with the least complications and high specificity and sensitivity are considered as the key points in establishing these programmes. In addition, recognising the problems of the community and important barriers in implementing the screening programme is considered as the important step.

Cost of colonoscopy, medications, insurance coverage and travel costs are regarded as the most common barriers reported in many previous studies [11, 18, 19]. These problems are common, even in the developed societies as the USA and European countries [24]. In the present study, the costs related to colonoscopy and laxative drugs and even pathology reports (in some cases) were paid from the project budget, as well as travel costs in the first phase of the study for those candidates referred from Azar Cohort population, which seems to be the most important facilitator and encouragement for people to participate in the study. Of course, this issue was resolved with the coordination and payment of costs in the first phase. Therefore, according to these people, it is more acceptable to have a comfortable technique or method which could be done at home, even with less accuracy diagnosis.

Based on the results of the present study, lack of knowledge and awareness of CRC screening among the population is considered as the most common barrier. Further, low public awareness about incidence, mortality and screening/early detection effectiveness was the major issue in this study. Therefore, PHC workers should be responsible for this aim in the front line of the society. In fact, it is necessary for general practitioners and specialists to provide people with such pieces of information correctly and regularly. In addition, physicians play a central role in individualised new information about the most effective techniques of CRC screening, and the importance and purpose of these modalities in reducing the burden of disease and mortality from advanced CRC [25–28].

Fear and discomfort are considered as other common problems among the participants. However, the term ‘fear’ had different meanings among the participants. Fear or worrying about colonoscopy and procedure tube, refereeing to the hospital and medical centre, finding any lesion or cancer and positive test results were the most common barriers in this study, respectively. In addition, lack of knowledge about curable cancers, fear of colectomy and heavy treatments were reported. Thus, some people prefer not to know at all if they have cancer, or at least be diagnosed later so that they can live more easily and without worries instead of dealing with cancer treatment and costs. Providing enough information about early diagnosis and screening programmes, curable early stages of CRCs and preventing deaths from CRCs were highlighted by PHC workers and health policy makers among the population [29–31].

Colonoscopy scheduling, crowded referral colonoscopy centres and being away from home were cited in most previous reports [28, 32]. In this study, some facilities including scheduling and facilitating regular visits in referral colonoscopy centres were provided. Involving about ten expert gastroenterologists and coordinating with four referral colonoscopy centres were considered as the main reasons for handling and reducing the effects of this barrier. Thus, the participants were not involved in scheduling of colonoscopy and for receiving the results or possible pathology reports, which contributed to the success of the programme in other similar experiences with the help of individuals in scheduling, and possibility of following and sharing their test results [7, 32]. On the other hand, involving both male and female gastroenterologist could solve the people’s problem and barrier in the same-gender preference, mostly among women.

The misconception of people about the colonoscopy procedure, as well as fear of complications, pain and bleeding was considered as another important issue, which is related to a lack of knowledge about procedure and the prevalence of these problems. All of these issues were cited in other similar studies as the most important and common barriers [33, 34]. Increasing people’s awareness and attitude with the aids of PHC providers’ physicians, social networks and more importantly policy makers is regarded as the most effective modalities for increasing social awareness about screening methods.

In most of the previous studies, some differences were observed between the problems and barriers mentioned by men and women. Women are usually more upset and worried about having colonoscopy, fear of tube and a sense of embarrassment and discomfort. However, most of the problems related to men included lack of awareness about screening goals and results. They believed that no screening and colonoscopy is necessary because of having no symptoms or problems. The lengthy procedure, the need for laxative bowel preparation and even going to the health centre were considered as the main barriers among males which made them upset [35, 36].

The main barrier can be considered from the viewpoint of psychosocial and socioeconomic factors, which can affect the success of screening programmes in many ways. Factors such as gender, age, marital status, higher education, and high-income, and residency (urban or rural) were the most common factors in a large number of previous reports [35, 37–39]. Urban people, as well as those with higher education and higher socioeconomic status, actually know more about the objectives and outcomes of screening programmes. However, they are aware of colonoscopy method, along with its pain and possible complications at the same time. However, rural people are satisfied with a correct and sufficient explanation better and faster than urban people who are satisfied with participating in the study.

Physician–patient relationship and physicians’ recommendation and information about the importance and necessity of screening contributed to the well-being and participation of volunteers in the screening programme. Acceptable and convenient communication with general population with any socio-economic status and educational level, appropriate and sufficient explanation about the benefits and goals of the project and gaining people’s satisfaction were effective in performing the study better. Conducting regular follow-up, reminding colonoscopy time and following the participants’ bowel preparation before colonoscopy played a role in overcoming the cases of failure. In some cases, we needed to call and follow-up them several times. Assisting and helping them patiently and calmly about their fears, embarrassment, worries and discomforts could easily encourage them to follow the screening programme. Furthermore, the way people are informed and invited, the cold and inappropriate attitude and the interest of co-investigators may lead to the failure of screening programmes [26, 27, 29, 38, 40, 41].

The latest COVID-19 pandemic led to the complete cessation in the present study. Fear of being contagious, contracting the infection by participants and the cessation of cooperation of research staffs and students were considered as the final and main barriers in this study. However, after consulting with the main co-investigators of the study, it is believed that we should stop screening due to the hazard of contagious from the hospital environment, which was the main place for screening for both the staff and participants. It seems that no similar organisation can continue its programme routinely during these circumstances.

### Limitations of the study

Facing with unavoidable complications during any intervention is considered as the main challenging problem in different studies. In this study, just one colonoscopy complication was emphasised as bowel perforation in the first phase of the study, which was considered as the main barrier leading to excluded cases. Sometimes, the main researcher had to be engaged and handle the psychosocial and economic burden and efforts of any complication.

A very good physician–patient relationship was observed in this study, as one of the main strengths of this project. However, providing sufficient information and test results, conducting proper follow-up and physicians’ examination were regarded as the main challenges for implementing screening programmes in any society. In addition, covering most expenses of the participants, the great job of well-trained research staffs and medical students in scheduling, following and providing the appropriate knowledge and attitude were considered as other strengths in this study.

Believing and preferring to be examined by the same gender physician was considered as one of the main problems by considering the religious and cultural conditions of the population where more than 99% of Iranians are Muslim. Therefore, the encouragement and cooperation of ten expert gastroenterologist physicians from both males and females in the study and the possibility of selecting a physician by the individual preference were highlighted in this study.

Recently, the start of the COVID-19 outbreak has been considered as the biggest barrier in this study leading to the practical shutdown of the research process. Since the colonoscopy centres in this study were referral treatment centres for COVID-19 patients simultaneously, we decided to stop the programme in spite of following all the safety points and sterilisation. In fact, about 98% of people who have had a colonoscopy schedule in recent months stopped to participate in this study because of worrying about developing coronavirus infection. Finally, with the start of the general quarantine programme, the closure of universities dormitories, our working medical students, who played a key role in persuading people to screen, stopped their cooperation.

## Conclusion

CRC screening is considered to be a complex programme leading to multiple problems and barriers in the form of socioeconomic and psychosocial factors in different societies and countries. Although these factors may be discussed based on system- and patient-related factors, discomfort, embarrassment, fear, lack of knowledge, costs and availability of techniques are the most important barriers in implementing screening programmes in populations due to the cultural and economic conditions of the societies.

Increasing knowledge and attitude, providing accurate and sufficient information at the population level, as well as improving communication and confidence between Physician–Patient and patient-health workers are considered to be the cornerstones of any population-based programmes. Further, the availability of screening techniques for age eligible population, as well as providing and introducing the best, simplest, cost-effective and noninvasive method based on a high sensitivity and specificity should be considered among the priorities of any organisations and policy maker. It is hoped that we can implement and establish CRC screening programmes in the near future in Iran, and decrease the incidence and mortality of CRC through detecting CRC as early as possible in the curable stages.

## Funding

The study was approved and supported as a research grant, by the Ministry of Health and Medical Education, Deputy of Research and Technology (Grant number: 700/98, 2015.03.14 [1394/12/24]) from the Iran Ministry of Health.

## Conflicts of interest

The authors report no conflicts of interest in this work.

## Authors’ contributions

MHS, RD, SD and MAJ: Performed substantial contributions to the conception and design of the work; the acquisition, analysis and interpretation of data for the work;

BS, MS and MN: Performed drafting the work and revising it critically for important intellectual content;

FF and HMA: Revised and approved the version to be published;

All the authors confirmed agreement to be accountable for all aspects of the work in ensuring that questions related to the accuracy or integrity of any part of the work are appropriately investigated and resolved.

The manuscript has been read and approved by all the authors, that the requirements for authorship as stated earlier in this document have been met and that each author believes that the manuscript represents honest work, if that information is not provided in another form.

## Availability of data and material

Data are openly available in a public repository that issues datasets with the responsibility of the corresponding author.

## Figures and Tables

**Table 1.** Demographic characteristics of the study participants (*N* = 725).

Variable		Frequency	Percentage (%)
Gender	Male	425	58.6
	Female	300	41.4
Age	<50	283	39
	≥50	442	61
Residency	Urban	288	39.7
	Rural	437	60.3
Education	High educated	374	51.6
	Moderate educated	215	29.7
	Low educated	136	18.7
Risk assessment	Average risk	180	24.8
	Moderate risk	351	48.4
	High risk	194	26.8

**Table 2.** Patient-related and practical and health system-related barriers and problems among all 725 participants in CRC screening, in East Azerbaijan, Iran.

Barriers and/or problems	Patient-related factors	Number (percentage within group)	Practical and health system-related factors	Patient-related factors	Number (percentage within group)
Lack of knowledge about screening	Male (*n* = 425)	247 (58%)	Lack of screening advices	Male (*n* = 425)	149 (35%)
	Female (*n* = 300)	204 (68%)		Female (*n* = 300)	141 (47%)
Lack of knowledge about significance and advantages of screening	Male (*n* = 425)	204 (48%)	Prefer private clinic	Male (*n* = 425)	111 (26%)
	Female (*n* = 300)	186 (62%)		Female (*n* = 300)	105 (35%)
No need for screening because of having no symptom	Male (*n* = 425)	285 (67%)	Lack of knowledge of staff and health-workers	Male (*n* = 425)	64 (15%)
	Female (*n* = 300)	162 (54%)		Female (*n* = 300)	66 (22%)
Lack of knowledge about preventability and treatability of CRC	Male (*n* = 425)	149 (35%)	Travelling problems	Male (*n* = 425)	98 (23%)
	Female (*n* = 300)	162 (54%)		Female (*n* = 300)	141 (47%)
Fear of colonoscopy	Male (*n* = 425)	149 (35%)	Traveling costs	Male (*n* = 425)	43 (10%)
	Female (*n* = 300)	144 (48%)		Female (*n* = 300)	42 (14%)
Anxiety about going to health centres	Male (*n* = 425)	102 (24%)	Time-consuming and lengthy procedure	Male (*n* = 425)	204 (48%)
	Female (*n* = 300)	111 (37%)		Female (*n* = 300)	69 (23%)
Fear and discomfort of colonoscopy	Male (*n* = 425)	183 (43%)			
	Female (*n* = 300)	195 (65%)			
Embarrassment and dignity	Male (*n* = 425)	149 (35%)			
	Female (*n* = 300)	186 (62%)			
Failed to bowel preparation	Male (*n* = 425)	153 (36%)			
	Female (*n* = 300)	84 (28%)			
